# Increased expression of the immunosuppressive interleukin-35 in patients with non-small cell lung cancer

**DOI:** 10.1038/s41416-019-0444-3

**Published:** 2019-04-08

**Authors:** Lisanne Heim, Katerina Kachler, Raphaela Siegmund, Denis I. Trufa, Susanne Mittler, Carol-Immanuel Geppert, Juliane Friedrich, Ralf J. Rieker, Horia Sirbu, Susetta Finotto

**Affiliations:** 1 Department of Molecular Pneumology, Friedrich-Alexander-Universität Erlangen-Nürnberg (FAU), Erlangen, Germany; 2 Department of Thoracic Surgery, Friedrich-Alexander-Universität Erlangen-Nürnberg (FAU), Erlangen, Germany; 3 Institute of Pathology, Friedrich-Alexander-Universität Erlangen-Nürnberg (FAU), Erlangen, Germany

**Keywords:** Lung cancer, Oncogenesis, Surgical oncology

## Abstract

**Background:**

The immunosuppressive role of the cytokine IL-35 in patients with non-small cell lung cancer (NSCLC) is poorly understood. In this study, we analysed the localisation and regulation of IL-35 in the lung of patients with non-small cell lung cancer (NSCLC) to further elucidate the immune-escape of cancer cells in perioperative course of disease.

**Methods:**

Interleukin 35 (IL-35) was measured by ELISA in postoperative serum from 7 patients with NSCLC as well as 8 samples from healthy controls. Immunohistochemistry, FACS analysis, real-time PCR, as well as western blot from samples of the control (CTR), peri-tumoural (PT) and the tumoural (TU) region of the lung derived from patients with NSCLC and 10 controls were performed.

**Results:**

Here we found elevated levels of IL-35 in the TU region as well as postoperative serum from patients with lung adenocarcinoma. Consistently, we found an increased expression of IL-35^+^Foxp-3^+^ cells, which associated with *ARG1* mRNA expression and decreased *TNFA* in the TU region of the lung of patients with NSCLC as compared to their CTR region. Furthermore, in the CTR region of the lung of patients with NSCLC, CD68^+^ macrophages were induced and correlated with IL-35^+^ cells. Finally, IL-35 positively correlated with TTF-1^+^PD-L1^+^ cells in the TU region of NSCLC patients.

**Conclusions:**

Induced IL-35^+^Foxp3^+^ cell numbers in the TU region of the lung of patients with NSCLC associated with *ARG1* mRNA expression and with TTF-1^+^PD-L1^+^ cells. In the tumour-free CTR area, IL-35 correlated with CD68^+^ macrophages. Thus inhibitors to IL-35 would probably succeed in combination with antibodies against immune checkpoints like PD-L1 and PD-1 currently used against NSCLC because they would inhibit immunosuppressive macrophages and T regulatory cells while promoting T cell-mediated anti-tumoural immune responses in the microenvironment as well as the TU region of NSCLC patients.

## Background

Lung cancer is the most common malignancy type of cancer for both men and women and is associated with high mortality. Treatment options include surgery, radiotherapy, and chemotherapy, as well as the use of immunomodulatory antibodies.^1–3^

Unfortunately, only 16% of lung cancer patients are diagnosed at early stage of the disease. The 5-year survival rate for lung cancer is 56% for cases detected when the disease is still localised (within the lungs). For distant tumours (spread to other organs), the 5-year survival rate is only 5%.^4,5^

Non-small cell lung cancer (NSCLC) represents approximately 85% of all lung cancer cases.^6^ The success of a therapeutic intervention is unfortunately limited. One reason could be that the tumour cell activates different immunosuppressive strategies in its environment, resulting in inhibition of an effector T cell anti-tumour immune response, which might vary depending on the genetic background of the host. Transforming growth factor beta (TGFβ) is produced by many lung adenocarcinoma (ADC) cell lines and it induces immunosuppression in its microenvironment,^2^ inhibiting the host immune response by activating forkhead box P3 (Foxp3) transcription factor in naive CD4^+^ T cells, thus imprinting the T regulatory (Treg) phenotype at the cost of a T helper type 1 (Th1)–interferon-gamma (IFNγ)-dominated anti-tumour immunoresponse.^7^

Interleukin (IL)-35 is an immunosuppressive cytokine involved in tumour immune escape because it induces Treg cells.^8–10^ IL-35 is a heterodimer of the IL-12 family of cytokine composed by IL-12p35 subunit and IL-27β subunit Epstein–Barr virus induced 3 (EBI3).^11^ IL-35 has been shown to exhibit immunosuppressive activities that are not present in the other members of IL-12 family.^12,13^ It is expressed primarily by antigen-presenting cells like tolerogenic dendritic cells.^14^ IL-35 can directly suppress effector T cell proliferation and function. It is also able to expand regulatory responses to promote tolerance to infections by generating a potent population of IL-35-producing inducible Tregs (iTr35).^15^ Other newly recognised cellular sources of IL-35 are CD8^+^ Tregs and B regulatory (Breg) cells.^16^ This function has been associated with its pro-tumoural function. By contrast, IL-35 can prevent autoimmune diseases. A better understanding of the expression and regulatory mechanisms of IL-35 will be beneficial to the development of novel immune therapies against these diseases.^11^

In this study, we analysed the tumoural (TU), peritumoural (PT), and control (CTR) lung region from the residual post-surgery resected lung tissue, as well as lung control tissues, and analysed serum of patients with NSCLC and those of healthy subjects to understand the role of IL-35 in this disease.

## Materials and methods

### Human subjects and study design

This prospective study was performed at the Friedrich-Alexander-Universität Erlangen-Nürnberg (FAU), Erlangen, Germany and was approved by the ethics review board of the University of Erlangen (Re-No: 56_12B; DRKS-ID: DRKS00005376).

Patients were selected within the framework of the thoracic surgery conference who were admitted with suspected or ascertained lung cancer (NSCLC). The clinical data including age, gender, and smoking status were collected by questionnaire before surgical intervention. Previous studies from our group and Supplementary Table S1 summarise some clinical data of the patients.^7,17–19^

The diagnosis of lung cancer was based on pathological confirmation, and histologic types of lung cancer were classified according to the World Health Organisation histologic classification formulated in 2004. The staging of lung cancer was based on the Cancer TNM Staging Manual formulated by the International Association for the Study of Lung Cancer in 2010. As defined after chest CT scan analysis and macroscopic pathology, after surgery we de-limited and separated three lung regions: the TU area (TU: solid tumour tissue), the PT area (PT: up to 2 cm away from the solid tumour), and the tumour-free control area (CTR: >5 cm away from the solid tumour) (Fig. S1A). In all the selected patients, the surgery consisted of lung resection and lymphadenectomy (removal of regional mediastinal lymph nodes). Lung samples were collected after surgery at the Thoracic Surgery department of the Friedrich-Alexander University (FAU) in Erlangen, directly processed at the adjacent Pathology Institute, and immediately transported, kept cold, to the department of Molecular Pneumology.

The obtained lung samples were separated for cell isolation, fluorescence-activated cell sorting (FACS) analysis, RNA extraction, immunohistochemistry (IHC), western blot, etc. (Fig. S1B). Tissue lung arrays were generated as previously described for NSCLC and control subjects (Tables S1 and S2).^19^ In addition, postoperative blood samples were collected with aim to measure IL-35.

### Total cell isolation and flow cytometric analyses of human cells

Human tissue samples were cut into small pieces using scalpels (1–3 mm^2^) followed by the preparation of single-cell suspensions using the Tumor Dissociation Kit, human (Miltenyi Biotec, Bergisch Gladbach, Germany) and the gentleMACS™ Dissociator (Miltenyi Biotec) according to the manufacturer’s instructions. The resulting cell suspension was centrifuged (10 min, 1000 rpm, 4 °C) and the supernatant was discarded. The cells were resuspended in 10 ml of hypotonic solution (Ammonium-Chloride-Potassium buffer (ACK)) and immediately centrifuged again (5 min, 1000 rpm, 4 °C) to quickly remove the ACK lysis buffer. This was followed by cell resuspension in 10 ml of phosphate-buffered saline (PBS) + EDTA + 1% penicillin/streptomycin (Pen/Strep) (PAA Laboratories, Cölbe, Germany) and centrifugation (10 min, 1000 rpm, 4 °C). Cells were finally resuspended in PBS + EDTA + 1% Pen/Strep + 5% foetal calf serum (FCS) (PAA Laboratories) and cell numbers were determined using trypan-blue staining. Cells were directly analysed by flow cytometry.

For flow cytometric analyses, 0.75–1 × 10^6^ total cells were incubated with the respective mix of surface antibodies dissolved in PBS and incubated for 30 min at 4 °C. Afterwards, cells were either directly analysed or further processed for staining with fluorochrome-conjugated antibodies against intracellular Foxp3. For intracellular staining, cells were fixed and permeabilised with Fixation/Permeabilization concentrate/diluent in accordance to the manufacturer’s protocol (eBioScience, San Diego, CA, USA). Antibodies used for flow cytometry are shown in Supplementary Table S3. Flow cytometric analyses were performed using FACS Calibur and FACS Canto II (BD BioScience). Data sets were evaluated by the Cell Quest Pro version 4.02 (BD BioScience) and Flow-Jo v10.2 (FlowJo, LLC, OR, USA) software.

### Immuno-histo-chemistry (IHC) of paraffin-embedded tissue sections

IHC was performed on paraffin-embedded sections of the lung tissue arrays. Before staining, paraffin was removed from the slides by incubation at 72 °C for 30 min and treated with Roti-Histol (Carl Roth, Karlsruhe, Germany) two times for 5 min. The tissue sections were then rehydrated by immersion in ethanol series with descending concentrations (100%, 95%, 70%) for 3 min each and in deionised water for 1 min. For heat-induced antigen retrieval, slides were placed into a rack containing 50 ml 1 mM Tris-EDTA buffer, which was transferred into a pressure cooker followed by incubation at 120 °C for 5 min. Slides were then cooled down for 30 min at room temperature followed by incubation for 1 min in deionised water. Tissue was surrounded with a hydrophobic barrier using a barrier pen. In a next step, slides were stained with the respective primary antibody against IL-35, Foxp3, or thyroid transcription factor 1 (TTF-1) (Table S4) using the ZytoChem-Plus AP Polymer Kit according to the manufacturer’s instructions of Zytomed (Zytomed Systems GmbH, Berlin, Germany). For IHC single stainings, nuclei were stained with haematoxylin solution (Carl Roth) and slides were covered with coverslips using Aquatex (Merck, Darmstadt, Germany). For IHC double staining, the second antibody against IL-35 or programmed cell death ligand-1 (PD-L1) (Table S4) was applied and detected according to the manufacturer’s instructions of the Dako EnVision Detection System Kit (Dako Deutschland GmbH, Hamburg, Germany). CD68 IHC was performed in cooperation with the Institute of Pathology at the FAU Erlangen-Nürnberg using a Ventana Benchmark (Roche Diagnostics, Mannheim, Germany). Negative controls were not treated with the primary antibody; the other steps remain the same. Stained slides were scanned using the digital slide scanner (Scan 150, 3D Histech Ltd, Budapest, Hungary) at the Institute of Pathology. Whole slide images were visualised by the CaseViewer software (Version 2.0, 3D Histech Ltd). CD68 single staining was quantified using the Definiens Tissue Studio 4.1 software (Definiens, Munich, Germany) while IL-35 and Foxp3 single stainings as well as Foxp3/IL-35 and TTF-1/PD-L1 double stainings have been evaluated using the ImgaeJ Cell Counter (Version 1.46, Bethesda, MD, USA).

### Quantitative real-time PCR (qPCR)

Total RNA was extracted from frozen tissue samples by using peqGold RNA Pure (Peqlab, Erlangen, Germany) according to the manufacturer’s instructions. One microgram of the resulting RNA was reverse-transcribed into cDNA via the RevertAid™ First Strand cDNA Synthesis Kit (Fermentas, St. Leon-Rot, Germany) according to the manufacturer’s protocol. Each qPCR reaction mix contained 15 ng of cDNA, 300 nM transcript-specific forward and reverse primer, and 2× SoFast™ EvaGreen® Supermix (BIO-RAD, Munich, Germany) in a total volume of 20 µl. qPCR primers were purchased from Eurofins-MWG-Operon, Ebersberg, Germany. The primer sequences are listed in Supplementary Table S5.

### Lung tumour cell line cultures

The human A549 cell line was purchased and authenticated, according to tissue type or gene mutation for cancer research, from the ATCC bank approximately 5 years ago. Mycoplasma contamination was detected using the Mycoplasma Detection Kit (Absource Diagnostics GmbH, Munich, Germany), according to the manufacturer’s protocol (latest date: Aug 9, 2016). On average, we performed ten cell passages between thawing and used in the described experiments. Cells were cultured in Ham’s F-12K medium (Gibco, Thermo Fisher Scientific, Langenselbold, Germany), supplemented with 10% of FCS, 1% Pen/Strep and 1% of L-Glutamin (L-Glu) (Gibco, Thermo Fisher Scientific) at 37 °C and 5% of CO_2_.

The human H520 lung tumour cell line was kindly provided by Dr. Paolo Ceppi from the Universitätsklinikum Erlangen-Nürnberg. The squamous cell carcinoma (SCC) cell line H520 was purchased from the ATCC bank in 2009 and checked for authentication via STR DNA profiling and mycoplasma contamination in 2015. On average, between the usage of cells and thawing, ten passages were performed. H520 cells were cultured in RPMI-1640 medium (Gibco, Thermo Fisher Scientific), supplemented with 10% of FCS, 1% Pen/Strep, and 1% of L-Glu at 37 °C and 5% of CO_2_.

For in vitro analyses, 3 × 10^5^ cells were cultured in 6-well plates. In all, 25 ng/ml of IL-35 (Peprotech) were added to cell culture and incubated for 48 h in triplicate. For cell harvest, medium was removed and cells were washed with 1 ml PBS. A total of 300 µl Trypsin-EDTA (Anprotec, Bruckberg, Germany) was added to each well and incubated for 2–5 min at 37 °C and 5% of CO_2_ to detach the cells. The enzymatic reaction was stopped by addition of the respective medium containing 10% FCS. Cell count was determined with trypan-blue staining.

For flow cytometric analysis, cells were washed with 1 ml PBS and centrifuged at 1500 rpm, 5 min at 4 °C. Cells were incubated with the respective antibody mix for surface staining at 4 °C for 30 min in the dark. Antibodies used for flow cytometry are shown in Supplementary Table S3. Flow cytometric analyses were performed by using FACS Canto II (BD BioScience). Data sets were analysed by Cell Quest Pro version 4.02 (BD BioScience) and Flow-Jo v10.2 (FlowJo, LLC, OR, USA).

### Protein extraction and western blot analyses

For protein extraction, lung tissue samples were lysed in RIPA buffer (Thermo Fisher Scientific, Waltham, MA, USA) with added inhibitor cocktail (Roche Diagnostics, Mannheim, Germany), followed by homogenisation using the SpeedMill PLUS (Analytik Jena, Jena, Germany) and innuSPEED lysis Tube P (Analytik Jena). After centrifugation (5 min, 3000 rpm, 4 °C), supernatants were incubated on ice for 45 min, followed by centrifugation (1 × 5 min, 3000 rpm, 4 °C; 1 × 45 min, full speed, 4 °C). Finally, protein concentration was calculated after using Bradford Assay (Protein Assay Dye Reagent Concentration, Bio-Rad, Munich, Germany). Western blot analysis to detect Foxp3 and β-Actin (Table S6) was performed with 50 µg of total lung protein. Quantification of Foxp3/β-Actin protein levels was performed using the AlphaView Software for FluorChem Systems (Biozym Scientific, Oldendorf, Germany) as previously described.^19^

### Enzyme-linked immunosorbent assay (ELISA)

ELISA technique was utilised to analyse the cytokine concentration in cell culture supernatants. IL-35 serum levels were determined using the LEGEND MAX™ Human IL-35 Heterodimer ELISA Kit according to the manufacture’s instructions (Biolegend).

### Statistical analysis

Differences were evaluated for significance (*p < 0.05, **p < 0.01, ***p < 0.001) by the Student’s two-tailed *t* test for independent events (Excel, PC). Graphs were created with GraphPad Prism, Windows. Correlations were examined by importing data, which needed to be correlated, in XY-tables of GraphPad Prism 7 software, diagramed it with linear regression curve, and performed the two-tailed Pearson correlation analysis to get the *r* and *p* value (*p < 0.05; **p < 0.01; ***p < 0.001).

## Results

### Increased immunosuppressive IL-35^+^ cells in the lung TU region in patients who suffered from ADC

In this study, we investigated the role of the immunosuppressive cytokine interleukin 35 (IL-35) in the lung of patients who underwent surgery because of NSCLC. The clinico-pathological characteristics of the patients are shown in Supplementary Table S1 and were recently reported.^7,17–20^ We first performed IHC on histological tissue arrays, which were generated by using lung tissues dissected from the tumoral (TU) region as well as the tumour-free control (CTR) region of the lung of patients with NSCLC (Fig. S1A).

**Fig. 1:**
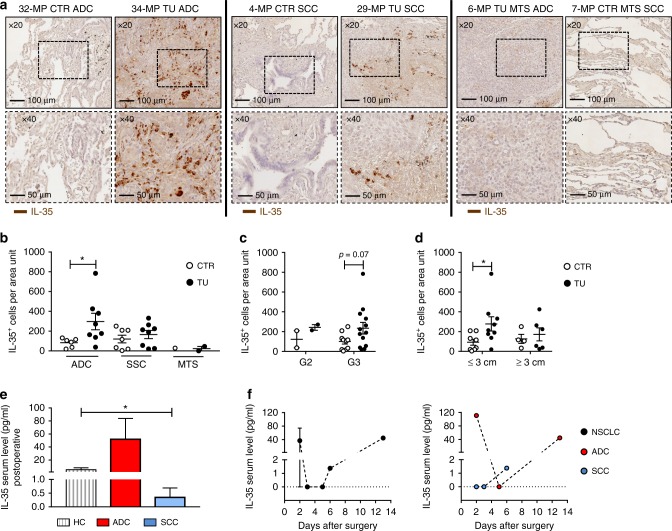
Increased production of interleukin (IL)-35 in the lung tumoural (TU) region of patients with adenocarcinoma (ADC). **a** Representative images of immuno-histo-chemistry (IHC) for IL-35 (brown) on paraffin-embedded tissue arrays from the control (CTR) and the TU region of the lungs of patients with ADC, squamous cell carcinoma (SCC), or metastatic lung cancer (MTS) (×20 and ×40 magnification). **b** Quantification of IL-35^+^ cells per area unit upon immunohistochemical staining (ADC_CTR_ = 6, ADC_TU_ = 8; SCC_CTR_ = 7, SCC_TU_ = 8; MTS_CTR_ = 1, MTS_TU_ = 2). **c**, **d** IL-35^+^ cells per area unit in the CTR and TU region of non-small cell lung cancer (NSCLC) patients categorised into grade 2 (G2) and grade 3 (G3) (**c**, G2_CTR_ = 2, G2_TU_ = 2; G3_CTR_ = 10, C3_TU_ = 13) and according to tumour diameters ≤3 cm and ≥3 cm (**d**, CTR_≤3 cm_ = 8, TU_≤3 cm_ = 9; CTR_≥3 cm_ = 4, TU_≥3 cm_ = 6). **e** Postoperative serum level of IL-35 detected by enzyme-linked immunosorbent assay (ELISA) in patients who suffered from ADC or SCC as well as from the lung of control patients without lung carcinoma (HC) (ADC = 3; SCC = 4; HC = 8). **f** Postoperative IL-35 serum level plotted over time (days after surgery) (right: NSCLC = 7; left: ADC = 3, SCC = 4). Data are presented as mean ± SEM and significance levels are indicated as follows: *p < 0.05

**Fig. 2:**
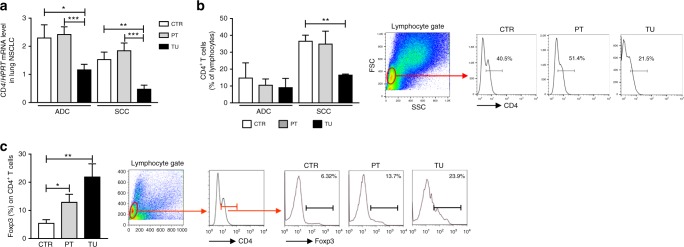
Decreased CD4^+^ T cell number and increased T regulatory cells expressing Foxp3 in the tumoural (TU) region of patients with non-small cell lung cancer (NSCLC). **a** Quantitative real-time PCR-based analysis of *CD4* mRNA expression in human lung tissue samples from the TU, peritumoural (PT), and control (CTR) region of patients suffering from adenocarcinoma (ADC) (ADC_CTR_ = 34, ADC_PT_ = 30, ADC_TU_ = 31) or squamous cell carcinoma (SCC) (SCC_CTR_ = 23, SCC_PT_ = 22, SCC_TU_ = 23) collectively grouped as NSCLC. **b** Flow cytometric analyses of CD4^+^ T cells (%) in total lung cell suspensions obtained from of the CTR, PT, and TU lung region of patients who suffered from ADC (ADC_CTR_ = 2, ADC_PT_ = 2, ADC_TU_ = 2) or SCC (SCC_CTR_ = 3, SCC_PT_ = 3, SCC_TU_ = 3) subtypes. **c** Flow cytometric analyses of Foxp3 in CD4^+^ T cells (%) in total lung cell suspension of the CTR, PT, and TU region of NSCLC patients (ADC_CTR_ = 4, ADC_PT_ = 4, ADC_TU_ = 4; SCC_CTR_ = 1, SCC_PT_ = 1, SCC_TU_ = 1). Representative dot plots of the gating strategy for CD4^+^ T cells and of Foxp3^+^ in CD4^+^ T cells are depicted (left, **b**, **c**). Data are presented as mean ± SEM and significance levels are indicated as follows: *p < 0.05, **p < 0.01, ***p < 0.001

In Fig. 1a, representative images of the IHC on histological lung tissue arrays with antibody to IL-35 are shown at different magnifications. We quantified the number of IL-35^+^ cells and found them upregulated in the TU region of patients with ADC as compared to the respective CTR region. Furthermore, analysis of metastatic ADC and SCC lung cancer patients did not reveal IL-35^+^ cells in the CTR or TU regions of the lung (Fig. 1b). We next asked whether the IL-35 expression is dependent on the histo-pathological grading. Tumours are defined as low or high grade (G), which is based on their degree of differentiation and growth rate (G1 = well differentiated, G2 = moderately differentiated—intermediate grade, G3 = poorly differentiated—high grade, G4 = undifferentiated—high grade) (Table S1). Well-differentiated tumours usually grow slowly, while poorly differentiated or undifferentiated tumours are characterised by an increased growth rate and metastasis. We found a trend towards upregulation of IL-35^+^ cells in the TU region of G3 lung cancer patients (Fig. 1c) indicating that IL-35 supports the metastatic spread of cancer cells at early stages. In addition, we found an upregulation of IL-35^+^ cells in the lung TU region of NSCLC characterised by a tumour diameter, as identified by computed tomography, <3 cm (Fig. 1d). By contrast, when the diameter size of the tumour was >3 cm, there was no IL-35^+^ cell number induction. Also, considering other classifications that take into account the tumour size, no significant changes in IL-35^+^ cells were detected (Fig. S1C, D). Taken together, these data might indicate increased tissue necrosis in the TU region of the lung at advanced stage of tumour development while IL-35 seems to play a role in the induction of metastasis of poorly differentiated high-grade tumours. In addition, we detected induced serum levels of IL-35 in postoperative blood samples from patients with ADC and decreased serum levels in patients with SCC as compared to healthy control subjects (Fig. 1e). We then started to follow-up IL-35 serum levels during postoperative time and observed a decreased level at days 2–4, followed by an induction of IL-35 to baseline level at day 13 (Fig. 1f), indicating a possible postoperative reduction of IL-35^+^ cells in the blood of patients with NSCLC. This study needs to be followed up in a bigger cohort of patients. Because IL-35 has been described to limit anti-tumour immunresponses, we next analysed *CD4* mRNA expression in a larger cohort of patients with NSCLC. We found a decreased expression of *CD4* mRNA in the TU region of patients with ADC and SCC, as compared to the respective CTR region as well as the PT region representing the tumour microenvironment of the lung (Fig. 2a, Fig. S1A). As IL-35 is increased in the TU lung region, these results indicate an immunosuppressive function of IL-35 on anti-tumour CD4^+^ T cell-mediated immune responses.

### Decreased CD4^+^ T cell number and increased Treg cells expressing Foxp3 in the TU region of patients with NSCLC

We recently described increased Foxp3^+^ Treg cells in the TU region compared to the PT and CTR area of NSCLC patients.^20^

To analyse immunosuppression in the TU region of these patients, we next determined the number of CD4^+^ T cells in isolated total lung cells by using FACS analysis. As shown in Fig. 2b, consistent with an immunosuppression in the TU region of these patients, by subdividing the patients with NSCLC into ADC and SCC, we found that patients with ADC are generally more immunosuppressed as compared to patients with SCC. In fact, in the control region of patients with SCC, a higher percentage of CD4^+^ T cells was detected as compared to ADC by trend. Moreover, we observed a significant downregulation of the percentage of CD4^+^ T cells in the TU region of patients with SCC as compared to their PT and CTR region. In contrast, CD4^+^Foxp3^+^ T cells were found induced in the TU region (Fig. 2c), indicating the presence of immunosuppression in the TU region of patients with NSCLC.

### Decreased expression of *IL12* cytokine family members in the TU region of patients with NSCLC

IL-35 belongs to the IL-12 cytokine family whose members are described as heterodimeric cytokines consisting of a α-chain (p19, p28, or p35) and a β-chain (p40 or p35). IL-35 is composed of EBI3 and p35. Furthermore, an interaction between EBI3 and p28 results in the formation of IL-27, whereas p35 in combination with p40 forms IL-12 (Fig. S2A).^21^ To investigate the regulation of IL-12 cytokine family members during NSCLC development, we next determined the expression of the different components in the TU, PT and CTR area obtained from patients who suffer from ADC and SCC (Fig. S2).

We found a trend towards decreased *IL12B*(p40) and a significant downregulation of *IL12A*(p35) expression in the TU region of both ADC and SCC patients (Fig. S2B, C), indicating an inhibition of IL12, at least on mRNA level, in the TU region of NSCLC patients.

### A direct correlation between *IL12A*(p35) and *EBI3* mRNA in the control and PT region of the lung of patients with ADC and SCC, respectively

We next analysed the correlation between the expression of *EBI3* and *IL12A*(p35), both part of IL-35, in the lung of patients with NSCLC (Fig. S3A, B). Here we found a direct correlation between *EBI3* and *IL12A*(p35) expression in the CTR region of patients with ADC (Fig. S3A) as well as in the PT region of patients with SCC (Fig. S3B). These data suggest that IL-35 heterodimer components are expressed at the mRNA level in the CTR and PT region of patients with ADC and SCC, respectively. Noteworthy, we found an upregulation of EBI3 in the PT region of the lung of these patients (Fig. S2E), indicating an important function of EBI3, and therefore IL-35 in the tumour microenvironment where not only T cell-mediated immune responses but also immunosuppression takes place. Further experiments in this direction are needed.

### *EBI3* correlates with *IL27*(p28) in the lung of patients with SCC

Because IL-35 was found induced in the TU region of ADC patients (Fig. 1a, b), we next investigated a possible preferential correlation between EBI3 and p28 to form IL-27 instead of IL-35 in SCC. Here we found a strong correlation between *IL27*(p28) and *EBI3* mRNA expression in all three regions of the lung of patients with SCC (Fig. S3C-E). These data indicate a preferential association of EBI3 with p28 to form IL-27 in the lung of patients with SCC.

### *EBI3* positively correlated with *CD4* in the lung of patients with NSCLC

We next asked whether *EBI3* mRNA expression would correlate with *CD4* in NSCLC. This point would give us an idea on whether EBI3 would contribute either to the immunosuppressive IL-35 or to the anti-tumour immune response as part of IL-27.^9,13^ In this analysis, we found a positive correlation between *EBI3* and *CD4* mRNA expression in the CTR region of patients with NSCLC (Fig. S4A, B) and in the PT region of the lung of patients with ADC but not in those with SCC (Fig. S4C, D). In the TU region of ADC patients, there was no association between *EBI3* and *CD4* (Fig. S4E). These data are consistent with immunosuppression and IL-35, which was found to be increased in the TU region of patients with ADC (Fig. 1b). By contrast, in the lung of patients with SCC, we detected a positive correlation between *EBI3* and *CD4* in the CTR and especially in the TU region of the lung (Fig. S4B, D, F). This latter finding is consistent with the presence of IL-27 rather than IL-35 in these patients. Thus this might suggest that patients with SCC have a better anti-tumour lung immune response as compared to patients with ADC.^22^

### IL-35^+^Foxp3^+^ T cells are induced in the TU region of patients with NSCLC and there they associate with TTF-1^+^PD-L1^+^ cells

IL-35 has been described to be an effective inhibitory cytokine produced by Treg cells and is required for their maximal suppressive capacity.^10,21,23^

Therefore, we next analysed whether the increased IL-35 production in the TU lung region could be associated with immunosuppression mediated by Foxp3^+^ Tregs.

**Fig. 3:**
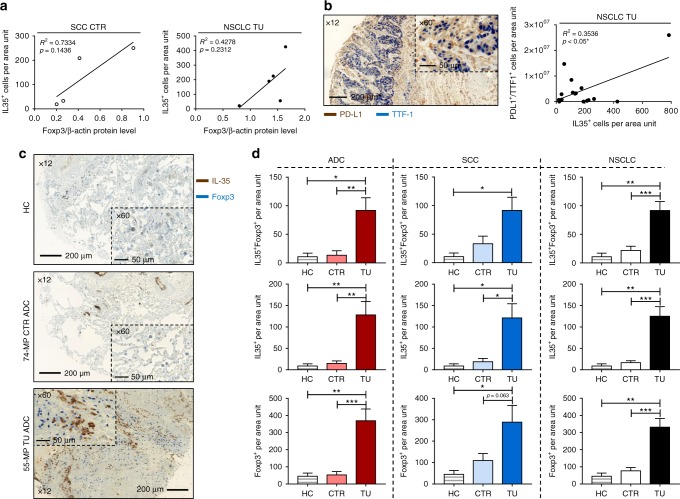
IL-35^+^Foxp3^+^ cells are induced in the tumoural (TU) region of patients with non-small cell lung cancer (NSCLC). **a** Correlation between IL-35^+^ cells per area unit detected via immunohistochemistry (IHC) and Foxp3/β-Actin protein levels quantified by western blot in the control (CTR) and TU area of patients with NSCLC (ADC_CTR_ = 0, ADC_TU_ = 1; SCC_CTR_ = 4, SCC_TU_ = 4). **b** Left: Representative images of double IHC for programmed cell death ligand 1 (PD-L1) (brown) and thyroid transcription factor 1 (TTF-1) (blue) on paraffin-embedded lung tissue from the TU region of a patient with ADC (×12 and ×60 magnification). Right: Correlation between IL-35^+^ and PD-L1^+^TTF-1^+^ double-positive cells per area unit detected via IHC in the TU (NSCLC_TU_ = 16) lung tissue of NSCLC patients. **c** Representative images of double IHC for interleukin (IL)-35 (brown) and Foxp3 (blue) on paraffin-embedded lung tissue from a control patient without lung carcinoma (HC) as well as from the CTR and TU region of patients with ADC (×12 and ×60 magnification). **d** Quantification of IL-35^+^Foxp3^+^ double-positive, IL-35^+^ single-positive, and Foxp3^+^ single-positive cells per area unit upon IHC staining of paraffin-embedded lung tissue arrays obtained from HC control patients (HC = 10) as well as from patients who suffered from ADC (ADC_CTR_ = 16, ADC_TU_ = 17) and SCC (SCC_CTR_ = 12, SCC_TU_ = 15) subtypes, collectively grouped as NSCLC. Data are presented as mean ± SEM and significance levels are indicated as follows: *p < 0.05, **p < 0.01, ***p < 0.001

In Fig. 3a, we correlated IL-35^+^ cell numbers detected via IHC (Fig. 1a) with Foxp3/β-Actin protein levels determined by western blot analysis (Fig. S5A, B). Here we found a trend towards positive correlation between IL-35^+^ cells and Foxp3/β-Actin protein levels in the CTR region of patients with SCC (Fig. 3a, right panel) as well as in the TU region of NSCLC patients (Fig. 3a, left panel). Furthermore, thyroid transcription factor 1 (TTF-1) is routinely tested in the diagnostic evaluation of suspected lung cancers. However, considering the recent success of anti-PD-L1 antibody immunotherapy for lung cancer,^17^ the significance of TTF-1^+^PD-L1^+^ cells needs further investigation.^24^ We thus asked whether TTF-1^+^programmed cell death ligand 1(PD-L1)^+^ cells in NSCLC would correlate with the presence of the immunosuppressive IL-35^+^ cells. Here we found a positive correlation between IL-35^+^ cells and TTF-1^+^PD-L1^+^ cells in the TU region of the lung of patients with NSCLC (Fig. 3b), whereas we did not find a correlation of these cells in the CTR region of the lung (Fig. S6C). Furthermore, double IHC for IL-35 and Foxp3 revealed an increased co-localisation of these two markers in the TU region of patients with NSCLC as compared to their CTR region as well as compared to control patients (Fig. 3c, d). Moreover, both IL-35^+^ and Foxp3^+^ cells analysed separately were found induced in the TU region of patients with NSCLC. In conclusion, immunosuppressive Foxp3^+^ cells were found associated with high levels of IL-35^+^ cells in the TU lung region of NSCLC patients.

### CD68^+^ macrophages are induced in the control region of patients with NSCLC where they correlated with IL-35^+^ cells

In this study, we also identified the PT area as the place of crosstalk between macrophages expressing *EBI3* and *p35* mRNA and the induction of IL-35^+^ cells in the TU region of NSCLC patients who might be relevant for future combined immunotherapy strategies.

**Fig. 4:**
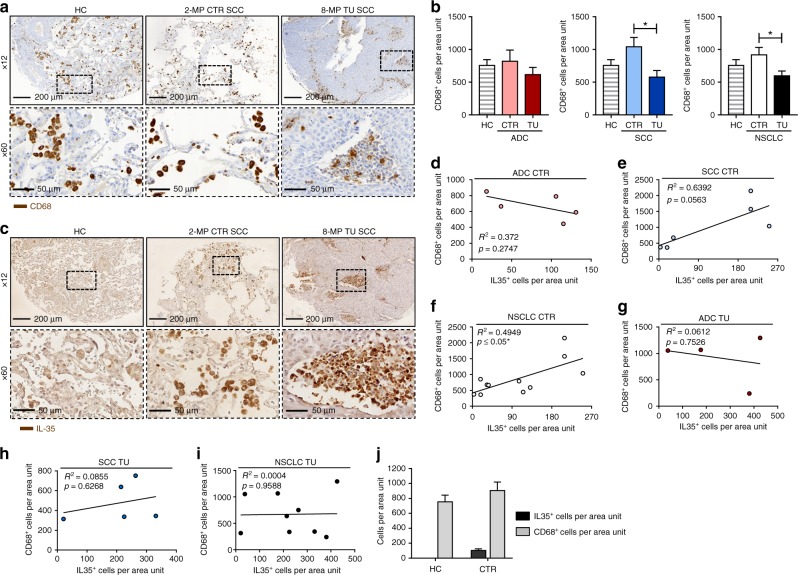
Interleukin (IL)-35 correlates with CD68^+^ cells in the control region of patients with non-small cell lung cancer (NSCLC). **a** Representative images of immunohistochemistry (IHC) for CD68 (brown) on paraffin-embedded lung tissue from a control patient without lung carcinoma (HC) and the control (CTR) and tumoural (TU) region of a patient who suffered from SCC (×12 and ×60 magnification). **b** Quantification of CD68^+^ cells per area unit upon IHC staining of paraffin-embedded tissue arrays from the lung of HC patients (HC = 10) as well as from the CTR and the TU region of the lung of patients with adenocarcinoma (ADC) (ADC_CTR_ = 18, ADC_TU_ = 12) or squamous cell carcinoma (SCC) (SCC_CTR_ = 14, SCC_TU_ = 10). **c** Representative images of IHC for IL-35 (brown) on paraffin-embedded lung tissue from a HC patient and the CTR and TU region of a patient who suffered from SCC (×12 and ×60 magnification). **d**–**f** Correlation between IL-35^+^ and CD68^+^ cells per area unit detected via IHC in the CTR lung tissue of ADC (**d**, ADC_CTR_ = 5) and SCC (**e**, SCC_CTR_ = 6) subtypes collectively grouped as NSCLC (**f**, NSCLC_CTR_ = 11) lung cancer. **g**–**i** Correlation between IL-35^+^ and CD68^+^ cells per area unit detected via IHC in the TU lung tissue of ADC (**g**, ADC_TU_ = 4) and SCC (**h**, SCC_TU_ = 5) subtypes collectively grouped as NSCLC (**i**, NSCLC_TU_ = 9) lung cancer. **j** Comparative depiction of IL-35^+^ and CD68^+^ cells per area unit of paraffin-embedded lung tissue of HC control patients without lung cancer (HC_IL-35+_ _cells_ = 10; HC_CD68+_ _cells_ = 10) and the tumour-free control region of NSCLC patients (CTR_IL-35+_ _cells_ = 13; CTR_CD68+_ _cells_ = 33) determined via IHC. Data are presented as mean ± SEM and significance levels are indicated as follows: *p < 0.05

We thus next analysed the localisation of macrophages in the lung of patients with NSCLC, as these cells are the main producers of the IL-12 family members IL-12, IL-23, and IL-27 and therefore possibly of IL-35.^21,25^ By using IHC for CD68 staining in our cohort of patients with NSCLC, we found a significant increase of these cells in the CTR region of the lung as compared to their TU region (Fig. 4a, b). In the CTR region but not in the TU region of the lung, CD68^+^ cells directly correlated with IL-35^+^ cells (Fig. 4d–i) in NSCLC in general and in SCC, specifically. Furthermore, IL-35^+^ cells in the CTR region of NSCLC patients resembled morphologically macrophages, which could not have been observed in lung tissue from control patients who do not suffer from lung cancer (Fig. 4a, c, j).

In conclusion, in the CTR region of the lung of patients with NSCLC, macrophages are induced in parallel with IL-35^+^cells, whereas in the TU region, IL-35^+^ cells correlated with Foxp3^+^ cells.^26,27^

### IL-35^+^ cells correlated with Arginase 1 (Arg1) mRNA expression in the TU region of patients with NSCLC

We next asked about the phenotype of the macrophages associated with the tumour environment and of those present in the CTR region of the lung and their relationship with IL-35-producing cells.^27^

**Fig. 5:**
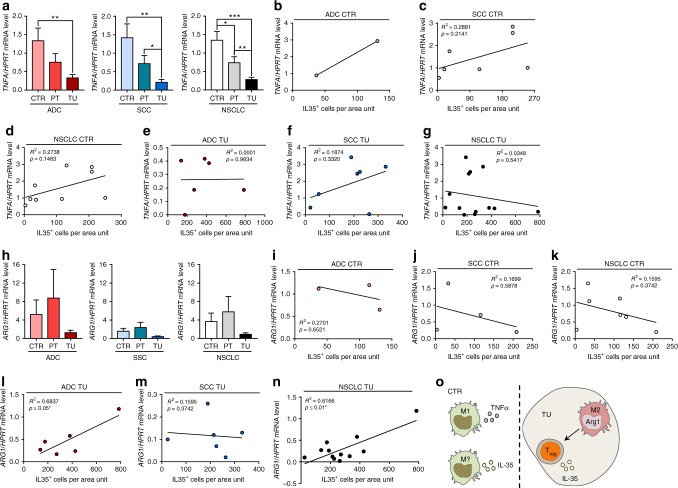
Correlation of interleukin (IL)-35 and Arginase-1 (Arg1)  in the tumoural (TU) region of non-small cell lung cancer (NSCLC) patients. **a** Quantitative real-time PCR (qPCR)-based analysis of *TNFA* mRNA expression in human lung tissue samples from the TU, peritumoural (PT), and control (CTR) region of patients suffering from adenocarcinoma (ADC) (ADC_CTR_ = 11, ADC_PT_ = 10, ADC_TU_ = 13) or squamous cell carcinoma (SCC) (SCC_CTR_ = 7, SCC_PT_ = 7, SCC_TU_ = 9) collectively grouped as NSCLC. **b**–**d** Correlation between *TNFA*/*HPRT* mRNA level and IL-35^+^ cells per area unit detected via immunohistochemistry (IHC) in the CTR lung tissue of ADC (**b**, ADC_CTR_ = 2) and SCC (**c**, SCC_CTR_ = 7) subtypes collectively grouped as NSCLC (**d**, NSCLC_CTR_ = 9) lung cancer. **e**–**g** Correlation between *TNFA*/*HPRT* mRNA level and IL-35^+^ cells per area unit detected via IHC in the TU lung tissue of ADC (**e**, ADC_TU_ = 6) and SCC (**f**, SCC_TU_ = 7) subtypes collectively grouped as NSCLC (**g**, NSCLC_TU_ = 13) lung cancer. **h** qPCR-based analysis of *ARG1* mRNA expression in human lung tissue samples from the TU, PT, and CTR region of patients suffering from ADC (ADC_CTR_ = 25, ADC_PT_ = 16, ADC_TU_ = 24) or SCC (SCC_CTR_ = 19, SCC_PT_ = 14, SCC_TU_ = 17) collectively grouped as NSCLC. **i**–**k** Correlation between *ARG1*/*HPRT* mRNA level and IL-35^+^ cells per area unit detected via IHC in the CTR lung tissue of ADC (**i**, ADC_CTR_ = 3) and SCC (**j**, SCC_CTR_ = 4) subtypes collectively grouped as NSCLC (**k**, NSCLC_CTR_ = 7) lung cancer. **l**–**n** Correlation between *ARG1*/*HPRT* mRNA level and IL-35^+^ cells per area unit detected via IHC in the TU lung tissue of ADC (**l**, ADC_TU_ = 6) and SCC (**m**, SCC_TU_ = 6) subtypes collectively grouped as NSCLC (**n**, NSCLC_TU_ = 12) lung cancer. **o** The immunosuppressive role of IL-35 in NSCLC: in the solid tumour, Arg1^+^ M2 macrophages attract IL-35-producing inducible Treg cells (iTr35), which support lung tumour growth. In the control region, a new macrophage subtype seems to secrete IL-35, which further promotes an immunosuppressive environment and supports the development of NSCLC. Data are presented as mean ± SEM and significance levels are indicated as follows: *p < 0.05, **p < 0.01, ***p < 0.001

Here we found that tumour necrosis factor alpha (TNFα), a marker of M1 macrophages, was found induced in the CTR region as compared to the TU region of the lung of patients with NSCLC (Fig. 5a). However, *TNFA* expression did not correlate with IL-35^+^ cells, neither in the CTR nor in the TU region of the lung of patients with NSCLC (Fig. 5b–g), indicating that the macrophages producing TNFα in the CTR region are not characterised by IL-35 production.

Moreover, Arg1, a marker of tumour-associated macrophages (TAMs) was found tendentially downregulated in the TU region of the lung of patients with NSCLC (Fig. 5h). In the TU but not in the CTR region of the lung, *ARG1* mRNA expression positively correlated with IL-35^+^ cells (Fig. 5i–n). These data indicate that the macrophages inducing IL-35^+^ cells located in the TU area of the lung of patients with NSCLC are likely to express *ARG1* mRNA and thus induce immunosuppressive cell-like Tregs (Fig. 5o). In the CTR region of the lung of patients with NSCLC, there are, probably, beside the classical M1 macrophages expressing TNFα, a second macrophage population that express IL-35 (Fig. 5o).

### IL-35 regulation of NSCLC cell survival

Because IL-35 was found elevated in the TU region of patients with ADC, we next asked whether IL-35 would influence the tumour cell survival (Fig. S7). By culturing both the ADC cell line A549 (Fig. S7A) and the SCC cell line H520 (Fig. S7C) with IL-35, we could not observe relevant changes in cell survival. We thus conclude that IL-35 does not influence lung tumour cell survival and death.

### IL-35 inhibited PD-L1 under metabolic deprivation in ADC tumour cell line

As described above and indicated in the literature, IFNγ, a potent anti-tumour cytokine, induced PD-L1 in A549 that points out to a double role of this cytokine in tumour because anti-PD-L1 trial has demonstrated to be successful in some cases of NSCLC.^7,28^ However, ADC is also signed by genetic imprints that must be considered, such as the expression of the epidermal growth factor receptor (EGFR) which binds to EGF, a potent growth factor for the ADC.^29–31^ Therefore, we next asked whether IL-35 would regulate these pathways under growth factor deprivation. Here we found that IL-35, as opposed to IFNγ, reduced PD-L1 but had no influence on EGFR, which remained upregulated under growth factor-deprivation conditions on A549 cells (Fig. S7B). By contrast, in SCC cell line under growth factor-deprivation conditions, reduced levels of PD-L1 were observed as compared to growth-favouring conditions. In this cell line, consistent with a less genetic involvement of this type of lung tumour, EGFR was relatively lower as compared to ADC and was not influenced by metabolic conditions (Fig. S7D).

Taken together, the data above indicate that the presence of IL-35 in the tumour cell microenvironment might influence the balance between EGFR and PD-L1 under metabolic restriction present in the tumour milieu of NSCLC.^27^

## Discussion

Recent success of immunotherapy against checkpoint inhibitors targeting PD-L1 and programmed cell death-1 (PD-1) molecules in lung cancer have shown some promising results.^32^ However, the success rate is still low, indicating the need of the combined immunotherapy to target the lung tumour growth dependent on the genetic background of the host. To this aim, we recently described the presence of a strong immunosuppressive microenvironment characterised by the expression of the Treg transcription factor signature gene Foxp3 induced by TGFβ that is secreted in the surrounding microenvironment of the lung tumour cells.^33^ IL-35 is a recently described cytokine that has been shown to have immunosuppressive function supporting Foxp3 in Treg and Breg cells, thus silencing the anti-tumour immune responses both in terms of cytokine and immunoglobulin production.^9,16^ We therefore set out to investigate in this study the regulation and expression of IL-35 in the lung of a cohort of patients with NSCLC. Here we found a difference in IL-35 regulation in patients with ADC as compared to SCC. In fact, in the CTR region of the lung of patients with ADC, IL-35 was expressed at lower level as compared to the respective TU region. In the SCC patients, there was also a trend towards IL-35 induction in the TU region, however, never reaching the statistical significance.

Confirming and extending this differential expression, in the serum of patients with ADC, IL-35 protein was found upregulated as compared to SCC and healthy controls. Also, immunosuppression in terms of CD4^+^ T cell inhibition was found more pronounced in the ADC CTR region as compared to SCC. The TU region, however, looks like a place where immunosuppression is high in both ADC and SCC patients.

Because IL-35, although immunosuppressive, is part of the IL-12 family, which is a cytokine family comprising anti-tumoural cytokines like IL-12 or IL-27,^21^ we started to look at its component regulation at the transcriptional levels. Here we found a direct correlation between *p35* and *EBI3* mRNA in the CTR and PT region of the lung of patients with ADC and SCC, respectively. However, *p35* and *EBI3* expression was found to be decreased in the TU region of ADC and SCC patients, indicating a downregulation of the mRNA components for IL-35 in the TU region where the cytokine was actually detected at the protein level. This apparent discrepancy could be due to the presence of not yet identified posttranscriptional regulatory mechanisms that need further investigations. Looking further at differences between ADC and SCC, we discovered that *EBI3* correlated very well with *p28* in all the three regions of the lung of patients with SCC, indicating a preferential association between EBI3 and p28 instead of p35 in patients with SCC.

Because IL-27 promotes Th1 T cell-mediated responses and IL-35 T cell immunosuppressive ones, we analysed the correlation between *EBI3* and *CD4* mRNA. Here *EBI3* mRNA was found positively correlated with *CD4* mRNA in the CTR region of the lung of patients with NSCLC. By contrast, a positive correlation was detected in the PT region of patients with ADC and in the TU region of SCC. To further address the question whether this correlation would lead to immunosuppression or anti-tumour immune responses, we looked at the correlation between IL-35^+^ cells and Foxp3^+^ T cells. IL-35^+^ cells tendentially correlated with Foxp3/β-Actin protein levels in the CTR region of SCC and in the TU region of NSCLC patients. In addition, by performing double IHC for Foxp3 and IL-35, we found an increase of double-positive IL-35^+^Foxp3^+^ cells in the TU region of patients with NSCLC. Owing to the resolution level of this technique, however, we cannot exclude the possibility that the co-localisation of these two stainings represent strict interaction between IL-35-producing cells such as macrophages and responding cells like Treg cells. At any rate, this represents strong evidence that IL-35 is linked to immunosuppression in the TU region of the lung of patients with NSCLC. In conclusion, given the higher levels of EBI3 in the PT region of patients with NSCLC, we think that this area is where IL-35 molecular components are assembled inside the macrophages. Possibly there might be a migration of these cells in the TU compartment where the IL-35 protein is assembled. However, implication that IL-35 is produced by TAMs is still indirect and thus further experiments are needed to confirm this point. To move forward towards these understanding, we stained sections for a macrophage marker CD68 and compared its localisation with IL-35 staining in lung section from patients with NSCLC. Here we found a decreased number of CD68^+^ macrophages in the TU region of the lung of patients with NSCLC and a positive correlation between CD68 and IL-35 in the CTR but not in the TU lung region. In contrast, only in the TU lung region, *ARG1* mRNA expression positively correlated with IL-35^+^ cells while there was a trend towards reduced *TNFA* expression with an increase of IL-35. Therefore, we concluded that, in the solid tumour, ARG1-positive M2 macrophages could attract or induce iTr35 cells, which support lung tumour growth. In the CTR region, a new macrophage subtype seems to secrete IL-35, which further promotes an immunosuppressive environment and supports the development of NSCLC. We next addressed the role of IL-35 on the regulation of NSCLC tumour cell survival and apoptosis. By using metabolic deprivation as well as addition of serum-supplemented medium, we could not detect any influence of IL-35 on the ADC lung tumour cell line A549 as well as on the SCC lung tumour cell line H520 with respect to cell survival or cell death. We further analysed the modulation of the checkpoint PD-L1 in the tumour cell lines cultured with IL-35. Although the ADC cell line expressed in general lower levels of PD-L1, in this cell line, IL-35 could reduce PD-L1 under growth factor-deprivation conditions. This mechanism is the opposite, as shown after incubation with IFNγ. By contrast, in the SSC cell line, IL-35 did not interfere with the downregulation of PD-L1 induced by serum starvation. Because EGFR is a very important marker for NSCLC, we then looked at EGFR regulation by IL-35. Under serum starvation conditions, EFGR was found induced in the ADC tumour cell line A549, whereas in the SSC cell line EGFR remained low no matter which metabolic condition was present.

Thus, in conclusion, besides TGFβ and IL-10, we have identified the presence and upregulation of the immunosuppressive cytokine IL-35 in the TU region of patients with ADC. We have also found that the PT region of the lung represents a location where EBI3 strongly maps in the lung of patients with ADC, and in this region we found a high association of EBI3 with IL-35^+^ cells. This situation is consistent with the finding in the autoimmune disease lupus erythematous, showing that EBI3^+^CD4^+^ cells were downregulated along with IL-35^+^ cells in these patients.^34,35^ In fact, autoimmune diseases as opposite to cancer are characterised by lack of immunosuppression.^36^

Current immunotherapy targets PD-L1 on tumour cells to activate tumour-infiltrating lymphocytes. We thus investigated the relationship of IL-35^+^ and TTF-1^+^PD-L1^+^ cells in the setting of lung cancer. Here we found a direct correlation between IL-35^+^ cells and TTF-1^+^PD-L1^+^ cells in the TU area, indicating that modern immunotherapy using anti-PD-L1/anti PD-1 antibodies should be analysed in combination with anti-IL-35 antibodies.

Thus IL-35 emerges as a new possible target to be associated with recent checkpoint inhibitors. Moreover, we described the possibility to easily measure this cytokine in serum immediately after lung surgery. Considering that our patients were analysed post-surgery and were not yet treated with radiation or other chemotherapeutical agents, this finding opens the possibility to set up personalised combined immunotherapy for NSCLC.

## Supplementary information

Supplementary Figures and Tables

## Data Availability

All data generated or analysed during this study are included in this published article and its supplementary information files.
