# Supplementary material for: Increased expression of the immunosuppressive interleukin-35 in patients with non-small cell lung cancer
Source: Br J Cancer. 2019 Apr 8;120(9):903–12. doi: 10.1038/s41416-019-0444-3 (PMC6734661; doi:10.1038/s41416-019-0444-3)
Supplement: Supplementary file 1 — Supplementary Figures and Tables [file 41416_2019_444_MOESM1_ESM.docx]

**Supplementary Figures and Tables**

**
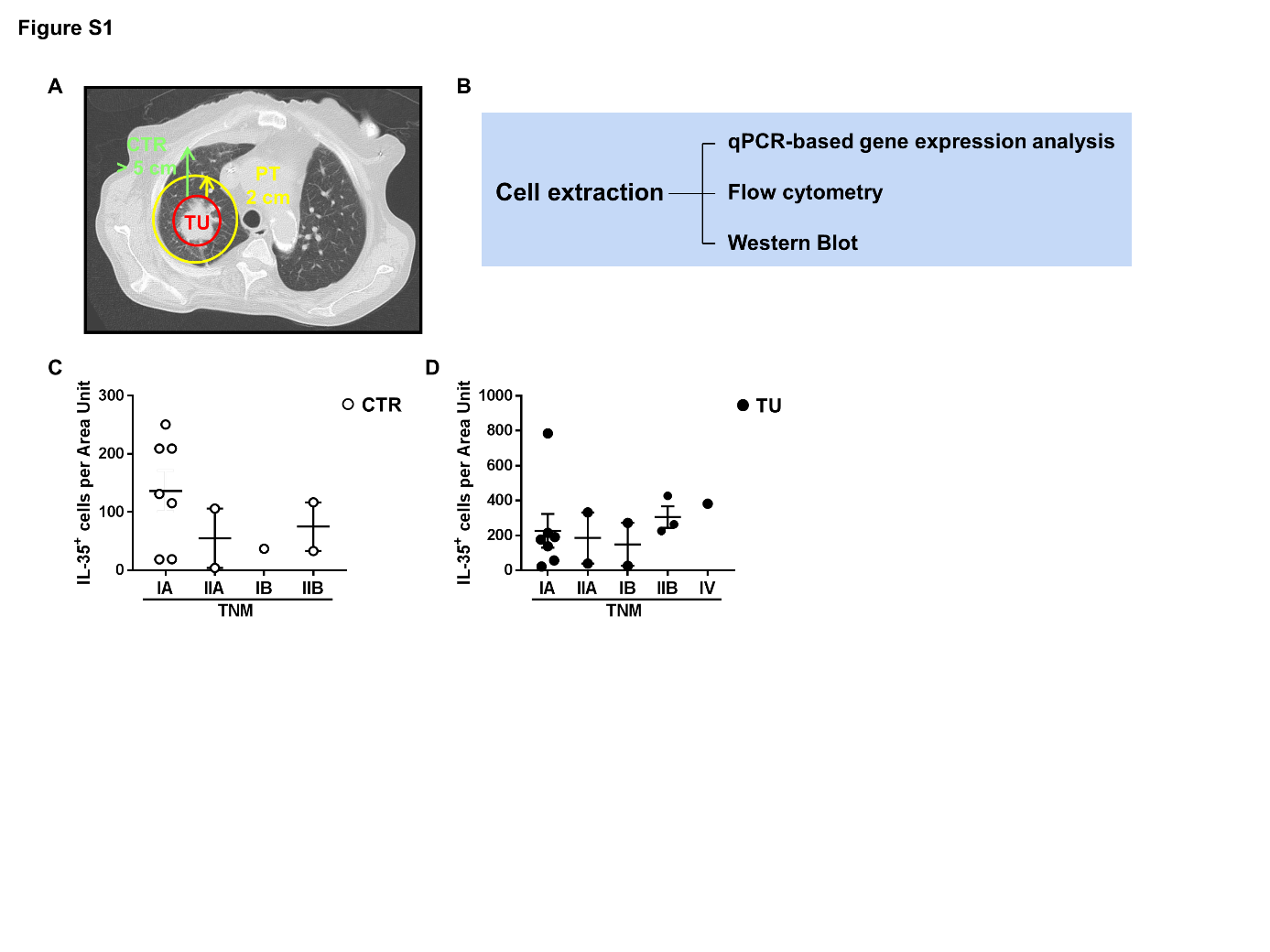
**

**Supplementary Figure 1: NSCLC patient study. (A)** Computed tomography image of the lung of a patient diagnosed with non-small cell lung cancer (NSCLC). After surgery, we obtained and processed lung tissue from three different lung regions: the tumour area (TU), representing the solid tumour tissue, the peri-tumoural area (PT) surrounding the tumour in a range of 2 cm and the control area (CTR), which is at least 5 cm away from the tumour border and free of tumour cells. **(B)** Total cells will be isolated from human lung tissue and used for RNA extraction and gene expression analyses or further analysed via immunohistochemistry (IHC), flow cytometry or western blot. **(C-D)** IL-35^+^ cells per area unit in the CTR (**C**, IA_CTR_=7, IIA_CTR_=2, IB_CTR_=1, IIB_CTR_=2) and TU (**D**, IA_TU_=7, IIA_TU_=2, IB_TU_=2, IIB_TU_=3, IV_TU_=1) region of NSCLC patients classified according to the TNM staging system. Data are presented as mean ± SEM.

**
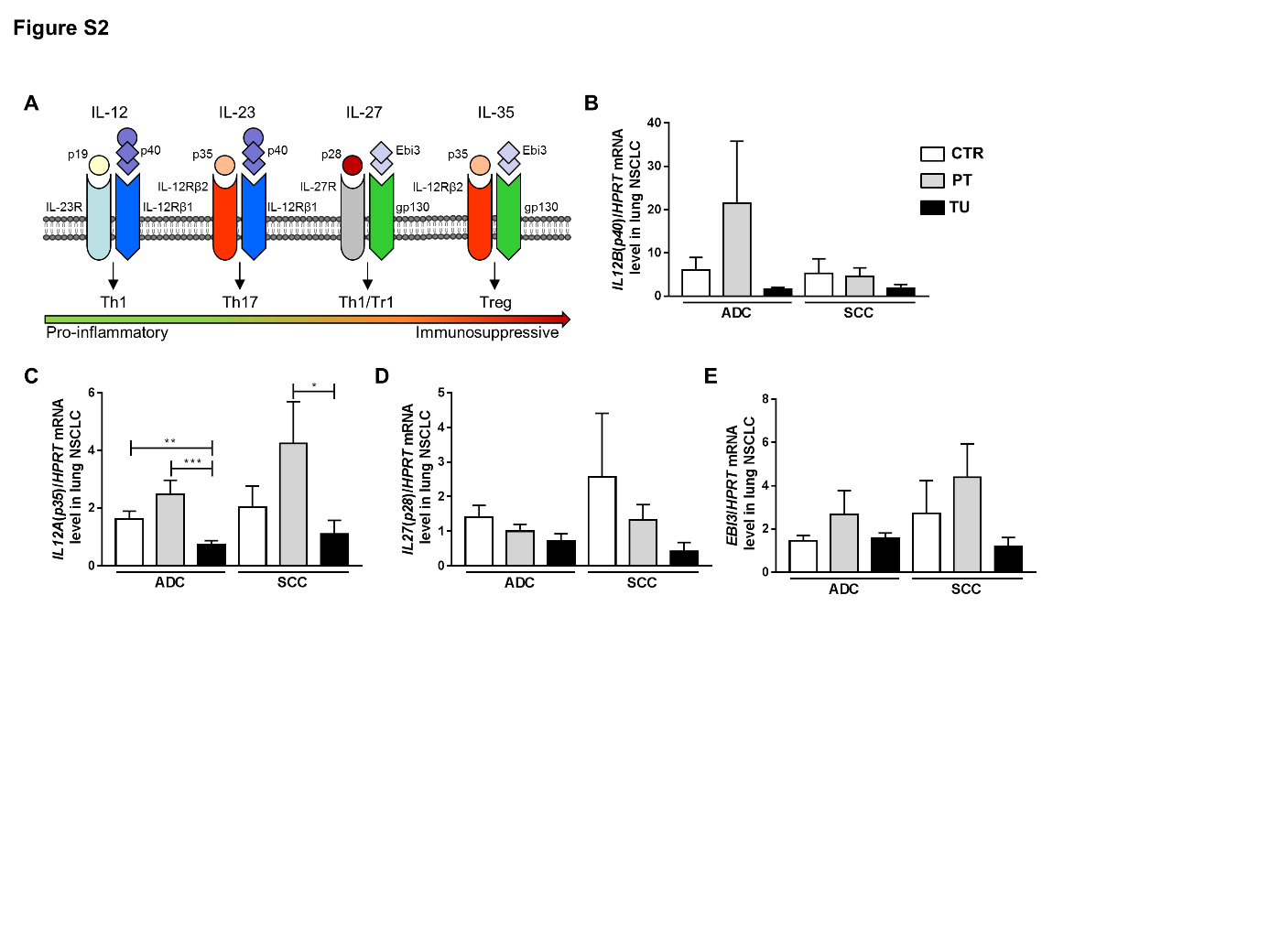
**

**Supplementary Figure 2: Decreased expression of IL12 cytokines in the TU region of patients with NSCLC. (A)** Structure of the IL-12 cytokine family. IL-12 family members are heterodimeric cytokines that consist of an α-chain (p19, p28 or p35) and a β-chain (p40 or EBI3). IL-12 is formed by the combination of p40 and p35 whereas IL-23 results from pairing between p40 and p19. Interaction between Ebi3 and p28 results in the formation of IL-27 while IL-35 is formed by pairing between Ebi3 and p35. These cytokines signal via different receptors. IL-12 induces signal transduction pathways via IL‑12Rβ1 and IL-12Rβ2 and IL-23 uses gp130 and IL-12Rβ2. Il-27 signals via gp130 and IL-27R while IL-35 uses gp130 and IL-12Rβ2. The function of IL-12 cytokines ranges from pro-inflammatory to immunosuppressive. IL-12 facilitates Th1 differentiation and the production of IFNγ whereas IL-23 is important for the development of Th17 cells. While IL‑27 has pro and anti-inflammatory functions by inducing the development of Th1 cells as well as of IL-10-producing Tr1 cells, IL-35 is an effective inhibitory cytokine produced by Treg cells. Furthermore, IL-35 promotes the development of iTr35 cells, a regulatory population of induced Tregs which mediates suppression via IL-35 but does not express Foxp3, IL-10 or TGFβ, adapted to Vignali and Kuchroo, 2012. **(B-E)** qPCR based analysis of *IL12B* (p40) (ADC_CTR_=29, ADC_PT_=26, ADC_TU_=25, SCC_CTR_=16, SCC_PT_=16, SCC_TU_=18) **(B)**; *IL12A* (p35) (ADC_CTR_=30, ADC_PT_=26, ADC_TU_=28, SCC_CTR_=17, SCC_PT_=17, SCC_TU_=18) **(C)**; *IL27* (p28) (ADC_CTR_=14, ADC_PT_=14, ADC_TU_=14, SCC_CTR_=8, SCC_PT_=6, SCC_TU_=8) **(D)** and *EBI3* (ADC_CTR_=32, ADC_PT_=25, ADC_TU_=29, SCC_CTR_=17, SCC_PT_=20, SCC_TU_=17) **(E)**. Data are presented as mean ± SEM and significance levels indicated as follows: *p<0.05, **p<0.01, ***p<0.001.

**
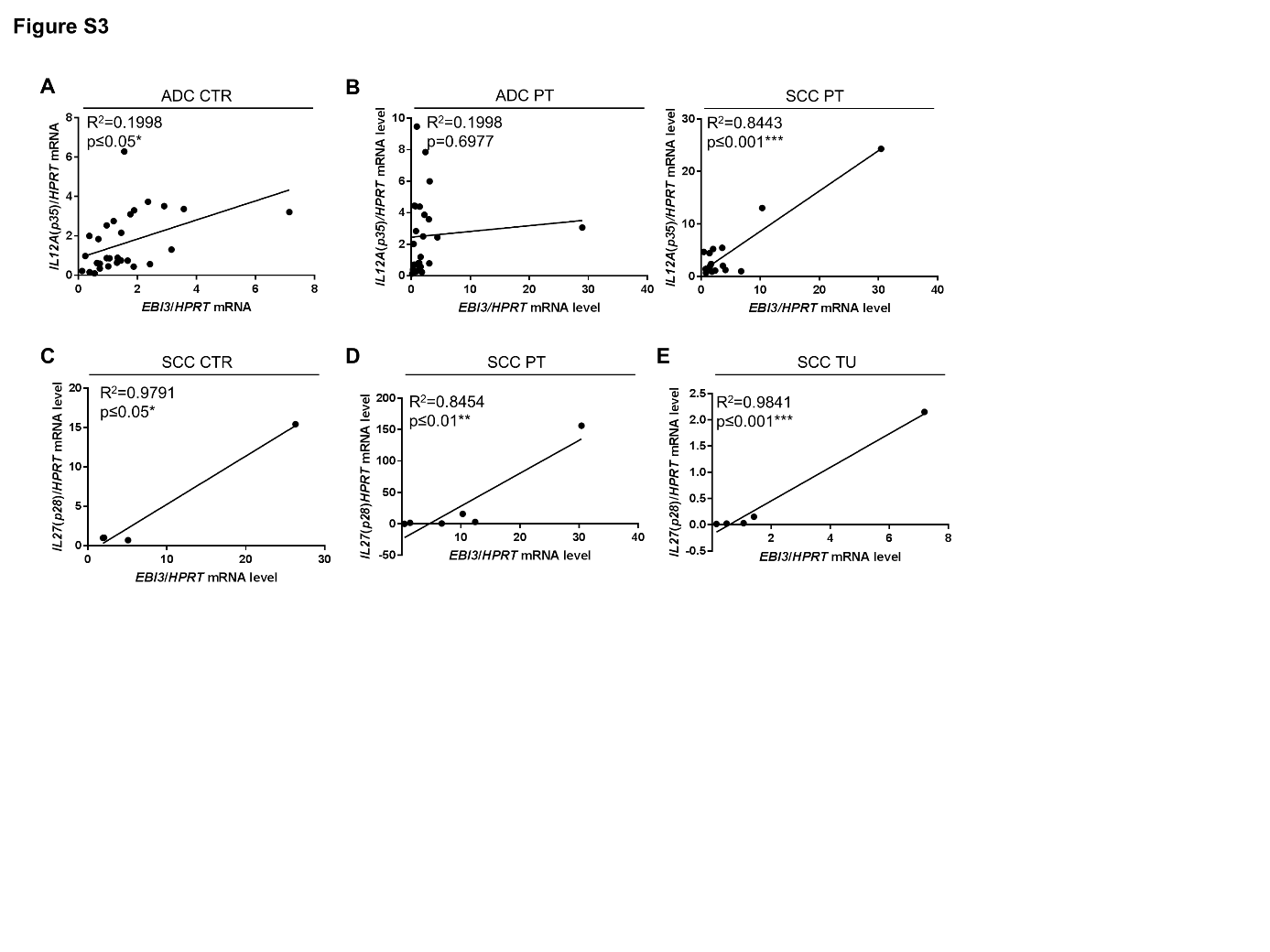
**

**Supplementary Figure 3: Correlation analysis between *EBI3* and *IL12A*(p35) as well as *IL27*(p28) mRNA expression. (A-E)** qPCR based correlation analysis of *IL12A*(p35)/HPRT to *EBI3/HPRT* in the CTR region of patients that suffered from ADC (ADC_CTR_=30) **(A)**; *IL12A*(p35)/HPRT to *EBI3*/*HPRT* mRNA expression in the PT region of NSCLC patients (left: ADC_PT_=25; right: SCC_PT_=17) **(B)**; *IL27*(p28)/*HPRT* to *EBI3*/*HPRT* mRNA expression in the CTR region of patients that suffered from SCC (SCC_CTR_=4) **(C)**; *IL27*(p28)/*HPRT* to *EBI3*/*HPRT* mRNA expression in the PT region of patients that were diagnosed with SCC (SCC_PT_=6) **(D)** and between *IL27*(p28)/*HPRT* and *EBI3*/*HPRT* mRNA expression in the TU region of patients that suffered from SCC (SCC_TU_=5) **(E)**. Correlations were examined by linear regression analysis as follows: *p<0.05, **p<0.01, ***p<0.001.


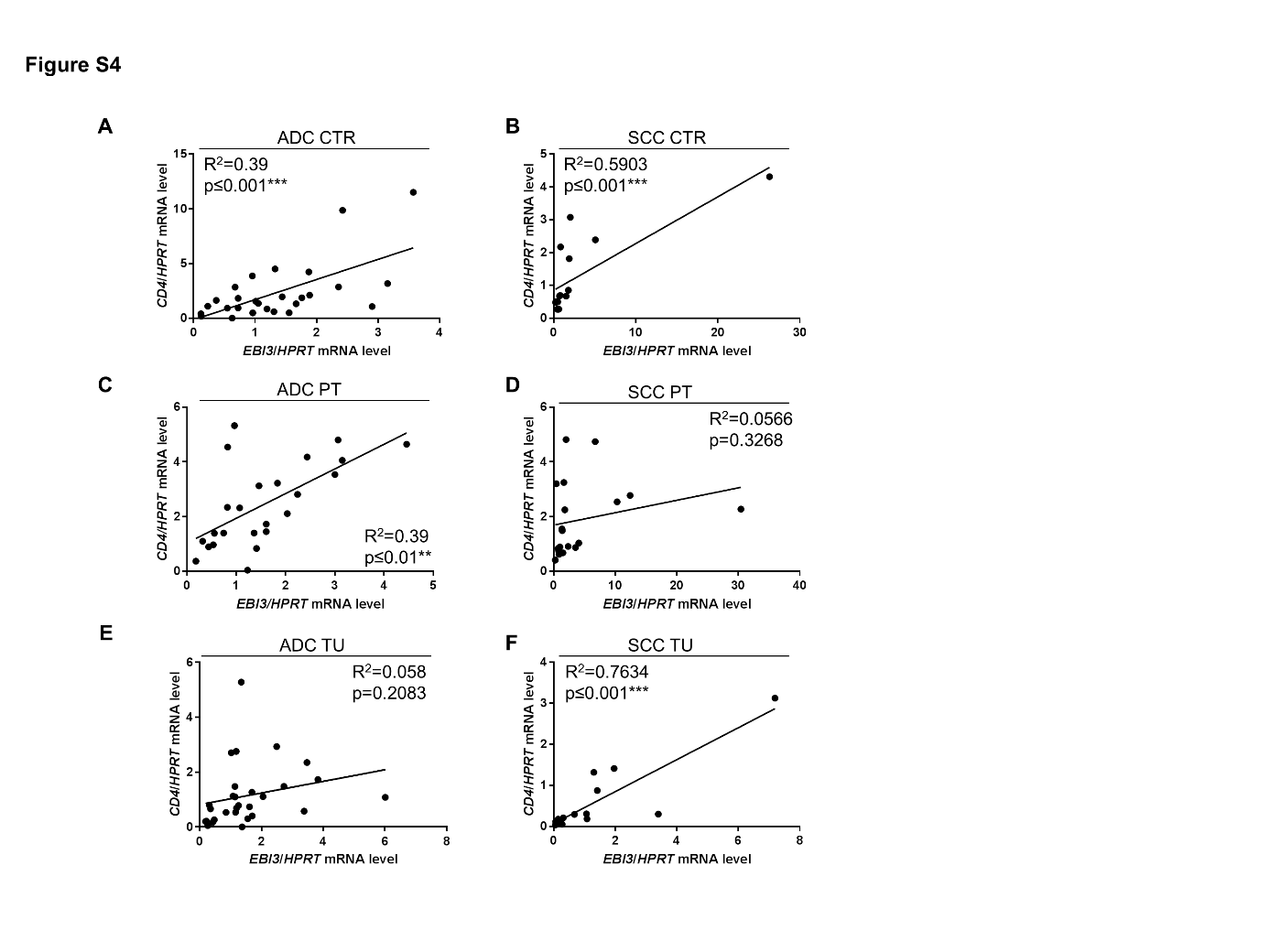


**Supplementary Figure 4: *EBI3* positively correlated with *CD4* in the lung of patients with NSCLC. (A-F)** Linear regression analysis of *CD4* and *EBI3* mRNA expression level relative to *HPRT*. Correlation analysis of *CD4/HPRT* to *EBI3/HPRT* in the CTR region of patients that suffered from ADC or SCC (ADC_CTR_=27, SCC_CTR_=15) **(A-B)**. Correlation analysis of *CD4/HPRT* to *EBI3/HPRT* in the PT region of patients that suffered from ADC or SCC (ADC_PT_=24, SCC_PT_=19) **(C-D)**. Correlation analysis of *CD4/HPRT* to *EBI3/HPRT* in the TU region of patients that suffered from ADC or SCC (ADC_TU_=29, SCC_TU_=16) **(E-F)**. Correlations were examined by linear regression analysis as follows: *p<0.05, **p<0.01, ***p<0.001.

**
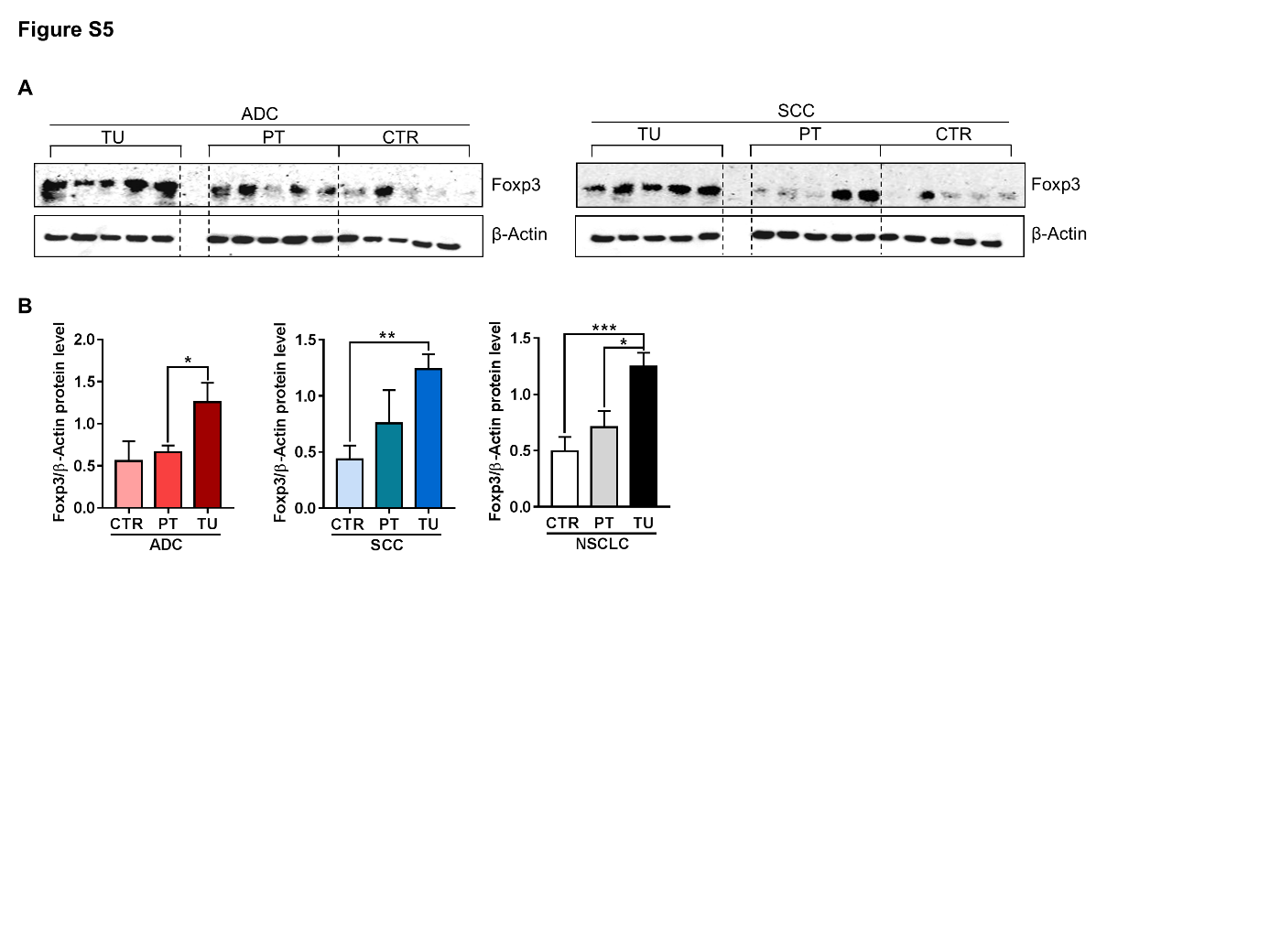
**

**Supplementary Figure 5: Foxp3 protein level in the tumoural, peri-tumoural and control lung region of NSCLC patients.**

**(A)** Western Blot of Foxp3 and β-Actin in the CTR, PT and TU area of patients with ADC (ADC_CTR_=5, ADC_PT_=5, ADC_TU_=5) or SCC (SCC_CTR_=5, SCC_PT_=5, SCC_TU_=5). **(B)** Respective Western Blot quantification of total Foxp3/β-Actin protein levels in the CTR, PT and TU area of patients that suffer from ADC and SCC subtypes, collectively grouped as NSCLC patients.


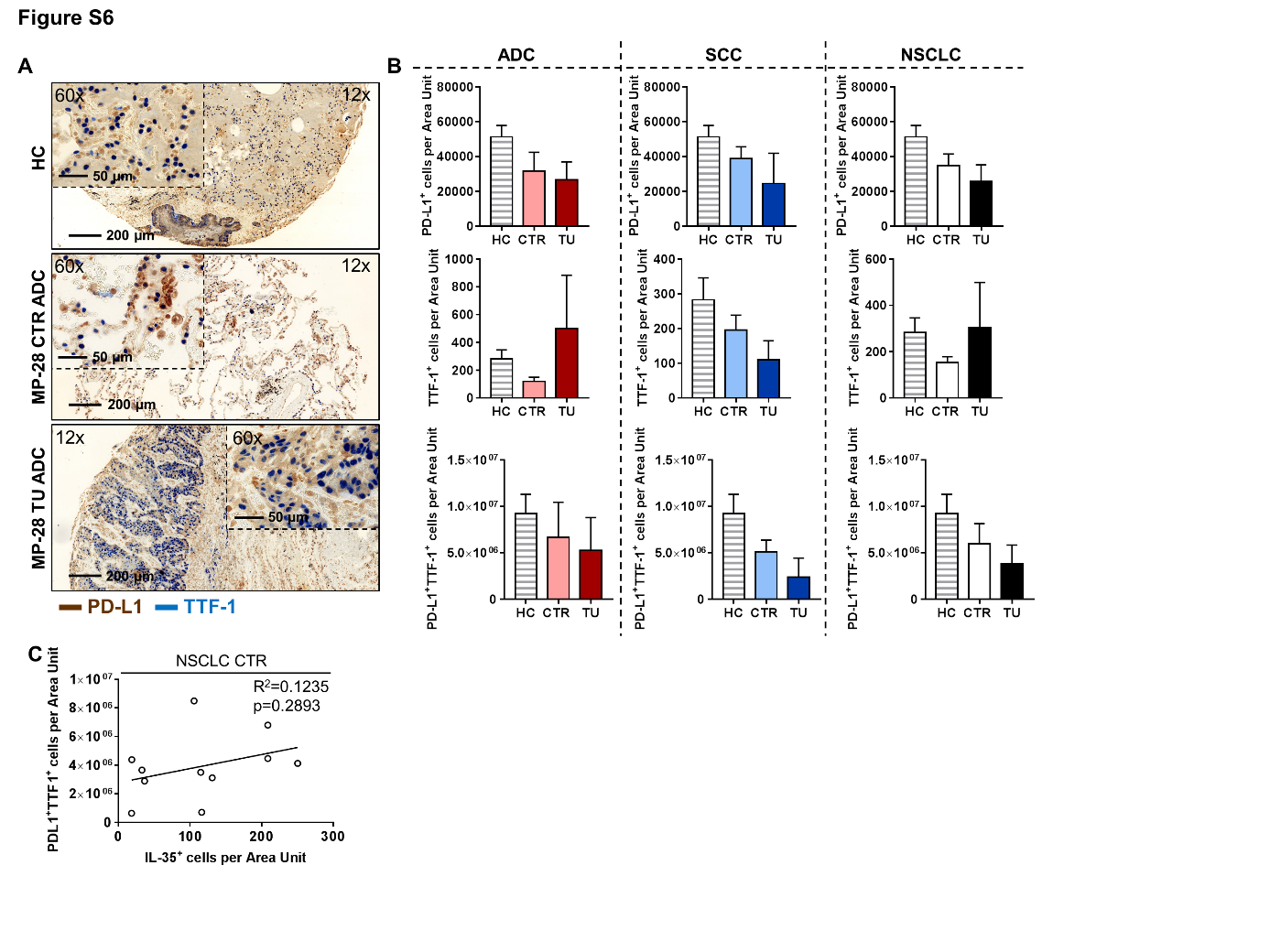


**Supplementary Figure 6: Correlation analysis between PDL1, TTF1 and IL-35.**

**(A)** Representative images of double IHC for PD-L1 (brown) and TTF-1 (blue) on paraffin-embedded lung tissue from a control patient without lung carcinoma (HC) as well as form the CTR and TU region of a patient with ADC (12x and 60x magnification). **(B)** Quantification of PD-L1^+^ single-positive, TTF-1^+^ single-positive and PDL-1^+^TTF-1^+^ double-positive cells per area unit upon IHC staining of lung tissue arrays obtained from HC control patients (HC=7) as well as from patients that suffered from ADC (ADC_CTR_=9, ADC_TU_=7) and SCC (SCC_CTR_=7, SCC_TU_=7) subtypes, collectively grouped as NSCLC. **(C)** Correlation between IL‑35^+^ cells and PDL-1^+^TTF-1^+^ double-positive cells per area unit detected via IHC in the CTR (**C**, NSCLC_CTR_=11) lung tissue of NSCLC patients. Data are presented as mean ± SEM. Correlations were examined by linear regression analysis as follows: *p<0.05, **p<0.01, ***p<0.001.

**
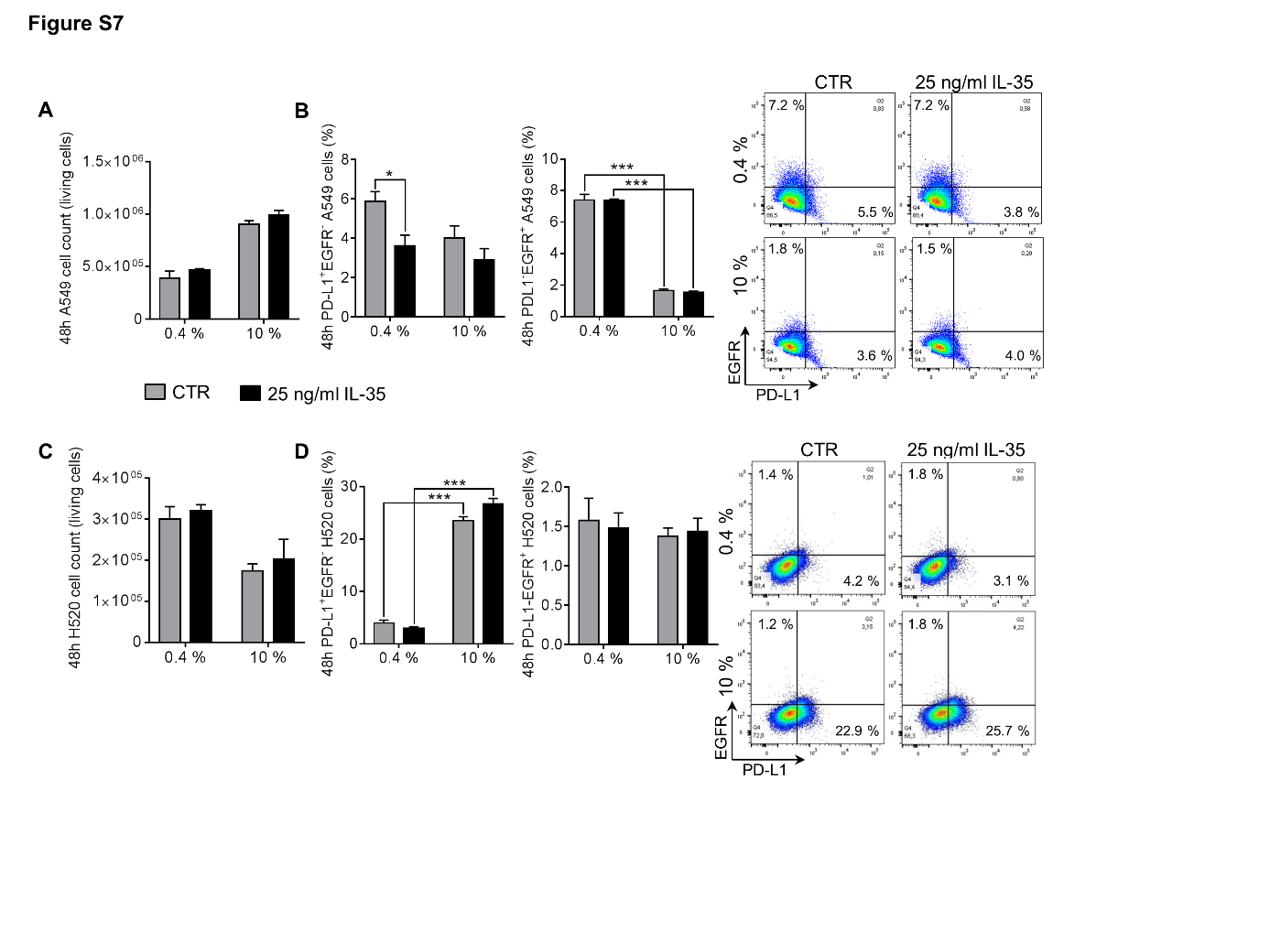
**

**Supplementary Figure 7: IL-35 regulation of NSCLC cell survival. (A-B)** A549 cells were cultured for 48h in medium supplemented with 0.4% or 10% FCS, in the presence or absence of 25 ng/ml IL-35 followed by the analysis of A549 living cell numbers **(A)** and by flow cytometry analysis of PD-L1 **(B, left)** and EGF-R **(B, right)** expression. **(C-D)** H520 cells were cultured for 48h in medium supplemented with 0.4% or 10% FCS, in the presence or absence of 25 ng/ml IL-35 followed by the analysis of H520 living cell numbers **(C)** and by flow cytometry analysis of PD-L1 **(D, left)** and EGF-R **(D, right)** expression. Experiments were performed with n=3. Data are presented as mean ± SEM and significance levels indicated as follows: *p<0.05, **p<0.01, ***p<0.001.

**Supplementary Table S1: Clinical patient data.**

| **Sample ID** | **Histological Classification** | **Tumour Ø (cm)** | **Grading** | **T** | **N** | **M** | **TNM Stadium** | **Gender** | **Age** | **Average Smoking (P/Y)** |
| --- | --- | --- | --- | --- | --- | --- | --- | --- | --- | --- |
| 1-MP | SCC | 1.3 | G3 | 1b | 0 | 0 | IA | Male | 80 | 40 |
| 2-MP | SCC | 5.1 | G3 | 1a | 0 | 0 | IA | Male | 57 | 40 |
| 3-MP | ADC | 5.0 | G3 | 2a | 0 | 0 | IB | Male | 79 | 60 |
| 4-MP | SCC | 2.0 | G3 | 1b | 1 | 0 | IIA | Female | 53 | 25 |
| 5-MP | SCC | 10.5 | G2 | 3 | 1 | 0 | IIB | Female | 67 | 50 |
| 6-MP | MTS-ADC | 4.0 | G3 | # | # | # | # | Female | 79 | 0 |
| 7-MP | MTS-SCC | 3.5 | G2 | # | # | # | # | Male | 45 | 1 |
| 8-MP | SCC | 5.5 | G3 | 3 | 0 | 0 | IIB | Male | 66 | 30 |
| 9-MP | ADC | 2.7 | G2 | 1b | 2 | 0 | IIIA | Female | 84 | 0 |
| 13-MP | SCC | 3.0 | G3 | 1b | 0 | 0 | IA | Male | 69 | 50 |
| 14-MP | SCC | 1.9 | G2 | 1a | 0 | 0 | IA | Female | 58 | 30 |
| 15-MP | ADC | 2.5 | G3 | 1b | 0 | 0 | IA | Male | 63 | 100 |
| 16-MP | ADC | 4.6 | G3 | 3 | 0 | 0 | IIB | Female | 70 | 15 |
| 17-MP | ADC | 2.6 | G2 | 2 | 0 | 0 | IB | Male | 74 | 70 |
| 19-MP | ADC | 6.5 | # | 2b | 0 | 0 | IIA | Female | 55 | 30 |
| 20-MP | ADC | 2.8 | G3 | 1b | 0 | 0 | IA | Male | 65 | 60 |
| 21-MP | SCC | 2.5 | G1 | 1b | 0 | 0 | IA | Male | 41 | 10 |
| 22-MP | ADC | 7.0 | G3 | 2b | 1 | 0 | IIB | Male | 68 | 82 |
| 23-MP | ADC | 4.5 | G2 | 2a | 0 | 0 | IB | Male | 73 | 75 |
| 26-MP | ADC | 1.3 | G3 | 1a | 0 | 1 | IV | Female | 52 | 50 |
| 27-MP | ADC | 1.4 | G3 | 1a | 0 | 0 | IA | Female | 70 | 50 |
| 28-MP | ADC | 1.2 | G3 | 1a | 0 | 0 | IA | Male | 76 | 60 |
| 29-MP | SCC | 3.7 | G3 | 1b | 0 | 0 | IIB | Male | 74 | 100 |
| 30-MP | SCC | 1.8 | G3 | 1a | 0 | 0 | IA | Female | 70 | 30 |
| 32-MP | ADC | 4.4 | G3 | 2a | 2 | 0 | IIIA | Female | 60 | 30 |
| 34-MP | ADC | 1.8 | G3 | 1 | 0 | 0 | I A | Female | 51 | 45 |
| 35-MP | ADC | 3.0 | G3 | 1b | 0 | 0 | IA | Female | 72 | 0 |
| 36-MP | SCC | 3.5 | G3 | 2a | 1 | 0 | IB | Male | 74 | 40 |
| 37-MP | SCC | 3.3 | G2 | 2a | 1 | 0 | IIA | Male | 60 | 45 |
| 39-MP | ADC | 6.0 | G3 | 2b | 0 | 0 | IIA | Male | 65 | 42 |
| 40-MP | ADC | 1.8 | G3 | 1a | 1 | 0 | IIA | Male | 82 | 100 |
| 41-MP | SCC | 1.3 | G3 | 3 | 1 | 0 | IIIA | Male | 70 | 42 |
| 42-MP | SCC | 1.5 | G3 | 2b | 1 | 0 | IIB | Male | 74 | 40 |
| 43-MP | ADC | 4.0 | G3 | 2a | 2 | 0 | IIIA | Female | 72 | 0 |
| 44-MP | ADC | 1.5 | G1 | 1a | 0 | 0 | IA | Male | 53 | 70 |
| 45-MP | ADC | 2.3 | G1 | 1b | 0 | 0 | IA | Male | 78 | 0 |
| 46-MP | SCC | 9.5 | G3 | 3 | 1 | 0 | IIIA | Male | 60 | 30 |
| 47-MP | SCC | 6.0 | G3 | 2b | 0 | 0 | IIA | Male | 64 | 80 |
| 48-MP | SCC | 5.0 | G2 | 2b | 0 | 0 | IIA | Male | 55 | 20 |
| 50-MP | SCC | 2.5 | G3 | 3 | 1 | 0 | IIIA | Male | 70 | 0 |
| 51-MP | ADC | 2.4 | G3 | 1b | 2 | 1 | IV | Male | 62 | 90 |
| 52-MP | ADC | # | # | # | # | # | # | # | # | # |
| 53-MP | ADC | 2.25 | G2 | 1a | 0 | 0 | IA | Male | 62 | 10 |
| 54-MP | SCC | 4.1 | G2 | 3 | 0 | 0 | IIB | Male | 72 | 0 |
| 55-MP | ADC | 1.8 | G3 | 1a | 2 | 0 | IIIA | Female | 64 | 40 |
| 56-MP | ADC | 4.0 | G2 | 2a | 0 | 0 | IB | Female | 67 | 0 |
| 57-MP | ADC | 3.8 | G2 | 2a | 0 | 0 | IB | Female | 35 | 10 |
| 58-MP | ADC | 6.5 | G3 | 3 | 0 | 0 | IIB | Female | 69 | 0 |
| 59-MP | ADC | 0.9 | G2 | 4 | 0 | 0 | IIIA | Male | 70 | # |
| 60-MP | SCC | 2.5 | G2 | 1b | 1 | 0 | IIA | Male | 71 | # |
| 61-MP | SCC | 1.1 | G2 | 1a | 0 | 0 | IA | Male | 75 | # |
| 62-MP | ADC | 3.5 | G2 | 1b | 0 | 0 | IA | Female | 80 | # |
| 63-MP | SCC | 9.0 | G3 | 3 | 1 | 0 | IIIA | Male | 69 | # |
| 64-MP | ADC | 3.5 | G2 | 1b | 0 | 0 | IA | Male | 55 | 35 |
| 65-MP | SCC | 2.8 | G3 | 1b | 0 | 0 | IA | Female | 76 | # |
| 68-MP | ADC | 8.0 | G3 | 3 | 0 | 0 | IIB | Male | 42 | 22 |
| 69-MP | ADC | 3.1 | G2 | 2a | 0 | 0 | IB | Female | 76 | # |
| 73-MP | ADC | 4.8 | G2 | 2a | 0 | 0 | IB | Female | 67 | 22 |
| 74-MP | ADC | 3.2 | G2 | 2a | 0 | 0 | IB | Female | 58 | # |
| 75-MP | SCC | 4.8 | G2 | 2a | 1 | 0 | IIA | Male | 54 | 35 |
| 76-MP | SCC | 3.1 | G3 | 2a | 0 | 0 | IB | Male | 65 | 40 |
| 77-MP | ADC | 0.9 | G2 | 1a | 0 | 0 | IA | Female | 64 | 45 |
| 78-MP | ADC | 2.1 | G3 | 1b | 2 | 0 | IIIA | Female | 80 | 0 |
| 79-MP | SCC | 10.0 | G4 | 3 | 0 | 0 | IIB | Female | 67 | 50 |
| 80-MP | ADC | 5.4 | G5 | 4 | 0 | 0 | IIIA | Male | 62 | 28 |
| 81-MP | ADC | 1.6 | G6 | 1a | 0 | 0 | IA | Male | 61 | 46 |
| 82-MP | SCC | 8.5 | G7 | 3 | 0 | 0 | IIB | Male | 82 | 24 |
| 83-MP | ADC | 5.5 | G8 | 3 | 0 | 1a | IVA | Female | 60 | 0 |
| 84-MP | SCC | 7.2 | G9 | 2b | 0 | 0 | IIA | Female | 55 | 45 |

Abbreviations: MP= molecular pneumology, ADC=adenocarcinoma; SCC=squamous cell carcinoma of the lung; MTS=metastasis; P/Y=Package/Year

Histopathological grading: G1=well differentiated; G2=moderately differentiated; G3=poorly differentiated

T-primary tumour: 0: No evidence of primary tumour; 1a: Tumour 2 cm or less in greatest dimension; 1b: Tumour more than 2 cm but not more than 3 cm in greatest dimension; 2a: Tumour more than 3 cm but not more than 5 cm in greatest dimension; 2b: Tumour more than 5 cm but not more than 7 cm in greatest dimension; 3: Tumour more than 7 cm

N-regional lymph nodes: 0: No regional lymph node metastasis; 1: Metastasis in ipsilateral peribronchial and/ or ipsilateral hilar lymph nodes and intrapulmonary nodes, including involvement by direct extension; 2: Metastasis in ipsilateral mediastinal and/or subcarinal lymph node(s)

M-distant metastasis: 0: No distant metastasis; 1: Distant metastasis

# No information available

**Supplementary Table S2. Clinical data of the cohort of control subjects analysed in this study.**

| **Sample**  **ID** | **Histological Classification** | **Tumour Ø (cm)** | **Grading** | **T** | **N** | **M** | **TNM Stadium** | **Average Smoking (P/Y)** |
| --- | --- | --- | --- | --- | --- | --- | --- | --- |
| K1 | Pneumothorax | 0 | / | / | / | / | / | # |
| K2 | Chondroid Hamartoma | 0 | / | / | / | / | / | # |
| K3 | Pneumothorax | 0 | / | / | / | / | / | # |
| K4 | Chondromatous Hamartoma | 0 | / | / | / | / | / | # |
| K5 | Pneumothorax | 0 | / | / | / | / | / | # |
| K6 | Rheumatoid Granuloma | 0 | / | / | / | / | / | # |
| K7 | Sarcoidosis | 0 | / | / | / | / | / | # |
| K8 | Pneumothorax | 0 | / | / | / | / | / | # |
| K9 | Chondroid Hamartoma | 0 | / | / | / | / | / | # |
| K10 | Pneumothorax | 0 | / | / | / | / | / | # |

Abbreviations: # No information available; / non-existing.

**Supplementary Table S3: List of antibodies used for Flow Cytometry.**

| Antigen | Fluorochrome | Clone | Supplier |
| --- | --- | --- | --- |
| CD4 | FITC | OKT4 | eBioscience, Inc., Frankfurt, Germany |
| EGF-R | BV510 | EGFR.1 | BD Biosciences, Heidelberg, Germany |
| PD-L1 | APC | MIH1 | BD Bioscience |
| Foxp3 | APC | 3G3 | Miltenyi Biotec, Bergisch Gladbach, Germany |

**Supplementary Table S4: List of antibodies used for immunohistochemistry (IHC).**

| Antigen | Clone | Dilution | Supplier |
| --- | --- | --- | --- |
| IL-35 | 15K8D10 | 1:20 | OriGene Technologies GmbH, Herford, Germany |
| Foxp3 | 236A/E7 | 1:50 | eBioscience |
| CD68 | PG-M1 | 1:200 | Dako, Deutschland GmbH, Hamburg, Germany |
| TTF-1 | 8G7G3/1 | 1:500 | Dako |
| PD-L1 | polyclonal | 1:100 | **Zytomed Systems GmbH, Berlin, Germany** |

**Supplementary Table S5: List of primers used for Quantitative Real-Time PCR.**

| Gene | Primer Sequence |
| --- | --- |
| *ARG1* | For: 5′-GAT TCC CGA TGT GCC AGG AT-3`  Rev: 5′-AAA TGT AGT GTT CCC CAG GGT C-3` |
| *HPRT* | For: 5′-TGA CAC TGG CAA AAC AAT GCA-3`  Rev: 5′-GGT CCT TTT CAC CAG CAA GCT-3` |
| *IL12A* (p35) | For: 5′-TCA GCA ACA TGC TCC AGA AG-3’  Rev: 5′-TAC TAA GGC ACA GGG CCA TC-3’ |
| *IL12B* (p40) | For: 5′-CAA GCA CTT CCT GAT GCA GA-3’  Rev: 5′-GCA GGA TTT CCT CAG CTG TC-3’ |
| *IL27* (p28) | For: 5′-GAG CAG CTC CCT GAT GTT TC-3’  Rev: 5′-AGC TGC ATC CTC TCC ATG TT-3’ |
| *CD4* | For: 5′-AAC CTG GTG GTG ATG AGA GC-3’  Rev: 5′-CTC AGC AGA CAC TGC CAC AT-3’ |
| *EBI3* | For: 5′-TGT TCT CCA TGG CTC CCT AC-3’  Rev: 5′-AGC TCC CTG ACG CTT GTA AC-3’ |
| *Foxp3* | For: 5′-AAC AGC ACA TTC CCA GAG TTC CT-3’  Rev: 5′-CAT TGA GTG TCC GCT GCT TCT-3’ |
| *PDL1* | For: 5′-AGC AAA GTG ATA CAC ATT TGG AG-3`  Rev: 5′-CCC CGA TGA ACC CCT AAA CC-3` |
| *TNFA* | For: 5′-CCC TGA AAA CAA CCC TCA GA-3`  Rev: 5′-AAG AGG CTG AGG AAC AAG CA-3` |

**Supplementary Table S6: List of antibodies used for Western Blot.**

| Antigen | Clone | Dilution | Supplier |
| --- | --- | --- | --- |
| Foxp3 | 236A/E7 | 1:100 | eBioscience |
| Β-Actin | I-19 | 1:800 | Santa Cruz Biotechnology, Inc., Heidelberg |
